# Cross-Linking Chitosan into Hydroxypropylmethylcellulose for the Preparation of Neem Oil Coating for Postharvest Storage of Pitaya (*Stenocereus pruinosus*)

**DOI:** 10.3390/molecules24020219

**Published:** 2019-01-09

**Authors:** Carmen G. Hernández-Valencia, Angélica Román-Guerrero, Ángeles Aguilar-Santamaría, Luis Cira, Keiko Shirai

**Affiliations:** 1 Laboratory of Biopolymers and Pilot Plant of Bioprocessing of Agro-Industrial and Food By-Products, Biotechnology Department, Universidad Autonoma Metropolitana-Iztapalapa, Av. San Rafael Atlixco No. 186, Iztapalapa, 09340 Mexico City, Mexico; em.carmenhdz@gmail.com (C.G.H.-V.); arogue@xanum.uam.mx (A.R.-G.); maas@xanum.uam.mx (Á.A.-S.); 2 Biotechnology and Food Science Department, Instituto Tecnologico de Sonora, 5 de febrero No. 818 sur, 85000 Obregon City, Sonora, Mexico; luis.cira@itson.edu.mx

**Keywords:** *Stenocereus pruinosus*, coating, chitosan, neem, azadirachtin

## Abstract

The market trend for pitaya is increasing, although the preservation of the quality of this fruit after the harvest is challenging due to microbial decay, dehydration, and oxidation. In this work, the application of antimicrobial chitosan-based coatings achieved successful postharvest preservation of pitaya (*Stenocereus pruinosus*) during storage at 10 ± 2 °C with a relative humidity of 80 ± 5%. The solution of cross-linked chitosan with hydroxypropylmethylcellulose with entrapped Neem oil (16 g·L^−1^) displayed the best postharvest fruit characteristics. The reduction of physiological weight loss and fungal contamination, with an increased redness index and release of azadirachtin from the microencapsulated oil, resulted in up to a 15 day shelf life for this fruit. This postharvest procedure has the potential to increase commercial exploitation of fresh pitaya, owing to its good taste and high content of antioxidants.

## 1. Introduction

*Stenocereus pruinosus* (Otto) Buxbaum is a columnar cactus endemic of the arid and semi-arid regions of Mexico, whose fruit is commonly named pitaya of May [1,2]. *S. pruinosus* widely distributes in thorn-scrub and tropical dry forests of Mexico, with harvesting from April to June. There are six identified variants of *S. pruinosus* fruit according to the colors of their pulp, which can be red, yellow, orange, purple, white, or pink. The pitaya fruit contains betalains, which are responsible for the flesh tonalities. Betalains are water-soluble and nitrogen-containing pigments as betaxanthin and betacyanins. Betaxanthins are in slightly higher concentrations than betacyanins in red flesh variants [3,4]. The betalains are chemopreventive, with several biological properties, such as anti-inflammatory, antioxidant, anti-diabetic, and anticancer properties [5,6]. Additionally, this fruit has a beneficial impact on human health due to a significant amount of phenolic compounds, vitamins B, C, and E, as well as minerals, such as iron, copper, and zinc [7,8].

It is worth mentioning that the market trend for pitaya is increasing due to the excellent acceptability of fresh consumption with nutritional similarities to other Cactaceae family fruit [9,10]. Nevertheless, the preservation of the quality of this fruit after the harvest is challenging, thereby it only has local distribution with significant decay after 3 days. Generally, the harvest index for the non-climacteric pitaya relies on color changes, epicarp brightness, or the ease of spines’ detachment rather than standardized quality-control procedures [1,8]. The common packing of the fruit in wooden crates causes damages and the release of juice, thus favoring microbial contaminations [1], and, moreover, there is a lack of information on the loss of the quality of pitaya due to microbial decay, dehydration, and oxidation. Armella et al. [1] report that pitaya (*Stenocereus griseus*, L.) loses attractive appearance and good taste after 5 and 8 days of storage at 17 and 7 °C, respectively. In another related work, García-Cruz et al. [4] report only 6 days of shelf life for this fruit at 24 °C. Additionally, the storage at low temperatures for pitaya (*Hylocereus undatus* and *Hylocereus polyrhizus*) as non-climacteric fruits harvested at close to full color retains market quality for at least 2 weeks at 14 °C or 1 week at 20 °C. However, these authors state the storage at 6 °C is counterproductive because of rapid losses of firmness and flavor when *Hylocereus* gets to room temperature [11].

Chitosan-based materials prove successful for increasing food shelf life, owing to its physicochemical characteristics and antimicrobial activity [12]. Chitosan (CH) forms adequate films with a semipermeable barrier for gas and moisture exchange control, modifying the endogenous level of CO_2_ and reducing ethylene in fruit. These characteristics render a delay in weight loss, maintain firmness and color in prickly pear fruit (*Opuntia*) when treated with CH solutions, notwithstanding the low scores for color, aroma, flavor, and overall acceptability after 15 days of storage [13]. A relevant feature is the chemical versatility that allows chemical bonding to other compounds, thus expanding its functionality. In this regard, the coating produced by the grafting of quercetin to CH was employed in *Opuntia ficus indica* to inhibit the enzymatic oxidation [14]. The combination of CH with hydroxypropylmethylcellulose (H) is often a strategy to improve performance on oxygen and lipid barrier properties [15]. Furthermore, the production of composites of CH and H with antimicrobial components, such as lysozyme and nanosilver, has been studied for the protection of meat from oxidative changes, spoilage, and growth of microorganisms [16]. Another approach is the application of a nanocomposite of CH-H with incorporated extracts of Neem oil (N) (*Azadirachta indica A. Juss*) films for grapes and plums’ packaging that extended postharvest shelf life up to 10 days by retarding enzymatic activity with adequate sensory and textural qualities [17]. Neem, originally from India, displayed antimicrobial properties ascribed to the active compound, azadirachtin, which is a limonoid tetranortriterpenoid, with applications in medicine or health products as well as in biopesticides [18]. The Neem has been used for the control of phytopathogenic fungi by Wang et al. [19], which describes the control of *Monilinia fructicola, Penicillium expansum, Trichothecium roseum*, and *Alternaria alternata* by Neem seed kernel extract in plums (*Prunus salicina*) and Yali pear (*Pyrus bertschneideri*). In another related study, Neem within 1.5% and 2% concentration coated precooling apples (*Malus domestica*) in a shrink-wrapped tray packing at 18–25 °C and with 65–75% relative humidity (RH) attained a 45 day storage life and remarkably reduced disease incidence with the minimum physiological weight loss [20].

Despite these studies on CH composites with H and Neem, there are constraints for many food applications as highly hydrophilic hydroxypropylmethylcellulose displays poor control of water permeability, thus favoring desiccation. To overcome this effect, the cross-linking of CH to hydroxypropylmethylcellulose by citric acid decreases the availability of H hydroxyl moieties, thus limiting polysaccharide-water interactions by hydrogen bonding and thereby reducing the water vapor transmission rate [15]. This study is the first to report, to the best of our knowledge, the preparation of CH cross-linked to cellulosic derivative for entrapping Neem and further assessment of its release in the postharvest storage of pitaya fruit. Moreover, the addition of the emulsifying Mesquite gum (MG) as a structural agent in this system enhances the preservation of this fruit. This study demonstrates that the use of these coatings on pitaya increases the shelf life, and thereby, the commercial exportation for this fruit. 

## 2. Results and Discussion

### 2.1. Characterization of Coatings

The molecular weights and degree of acetylation (DA) for CH define the range of applicability, thereby adhesion and moisture retention capacities, as well as film-forming abilities, relates to high molecular weights. In the case of the acetyl group, the lower the DA, the higher the antimicrobial activity throughout the ionic interaction of positively charged amine groups with the negatively charged phospholipids of the cell membrane. Interestingly, although the antimicrobial activity increases with low molecular weight CH, high molecular weights are effective against spore germination [21]. The CH sample of the present work by thermochemical deacetylation of biological chitin has a medium viscosimetric average molecular weight (Mv = 285.55 ± 0.19 kDa) and low DA of 9.91 ± 0.21% (Figure S1a shows the CH ^1^H-NMR spectrum).

On the other hand, the determination of the radical scavenging activity considers the variability among Neem commercial products. In this regard, the 2,2-diphenyl-1-picrylhydrazyl (DPPH) method on our sample displays an IC_50_ of 0.0178 ± 0.0006 g·L^−1^, which is lower than that by Nahak and Sahu [22] with 0.039 g·L^−1^. This variation is ascribed to the presence of other phenolic compounds and alkaloids in the oil. Besides, the concentration of azadirachtin for the preparation of emulsions in our Neem is 3.38 g·kg^−1^, and 16 g·L^−1^ (0.045 g·kg^−1^ of azadirachtin).

Entrapping matrices were hydroxypropylmethylcellulose grafted to CH (CHH) and CH mixed to MG (CHMG). CHH production was conducted throughout citric acid crosslinking using NaH_2_PO_4_ catalyst (Supplementary Figure S1 shows the scheme of the reaction) following the previous work by Alonso et al. [23]. ^1^H-NMR analyses displayed 74.12 ± 5.41% of hydroxypropylmethylcellulose crosslinked in CH (Supplementary Figure S1 shows ^1^H-NMR spectra for CH, hydroxypropylmethylcellulose, and CHH). The emulsion employed polyethylene glycol and d-sorbitol as plasticizers and tween 20, tween 80, and polyvinyl alcohol and MG as emulsifiers [24]. Table 1 shows a drop diameter (D_3.2_) for the emulsion of Neem in CHH solution (NCHH) significantly higher than that for emulsion of Neem in CH with added Mesquite gum (NCHMG), which points out to the crosslinking of the polymers, thus limiting the surface activity and the ability to absorb in the oil-water interface. On the contrary, the D_3,2_ for NCHMG suffers the influence of the surface activity of the Mesquite gum (MG) as a stabilizing agent. The biopolymers behave mainly as stabilizing agents on the surface charge of the emulsion drops (Figure S2 shows the ionic behavior of the polymers). Regarding the ζ, NCHH displays a positive surface charge, owing to the amino groups present in CH (Table 1), but negative for the NCHMG emulsion, owing to the MG carboxylic moieties (Table 1). Interestingly, NCHMG remains stable for one year and three months at room temperature. According to Acedo-Carrillo et al. [25], the stabilization of oil in water emulsions by MG might be explained for the electrosteric interaction of the protein-polysaccharide complexes thereof. Contrarily, NCHH emulsion readily creams after 45 min.

### 2.2. Effect of Coatings on Pitaya (S. pruinosus) Postharvest Quality Endurance

#### 2.2.1. Weight Loss (WL)

**Figure 1:**
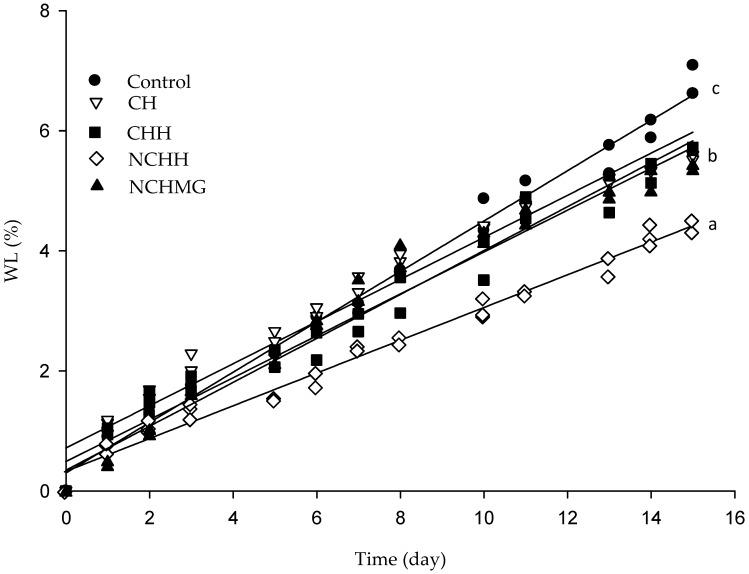
Weight Loss (WL) of pitaya (*S. pruinosus*) (*n* = 9) during storage at 10 °C and relative humidity (RH) of 80% (See Table 2 for treatment identification). Different letters among traces mean significant differences (*p* < 0.05).

WL rates fitted the experimental data of WL for each treatment and control in a second-order polynomial model with R^2^ > 0.98 (Figure 1, Table 2). Fruits having CH, CHH (control CH-*g*-hydroxypropylmethylcellulose), and NCHMG display WL rates of 0.55 ± 0.03 day^−1^, 0.38 ± 0.06 day^−1^, and 0.58 ± 0.04 day^−1^, respectively. All treated fruit has significantly different WL rates compared to the control (0.42 ± 0.03 day^−1^) and NCHH stands for the best treatment of 0.29 ± 0.07 day^−1^ rates.

In a related work, the WL of two variants of non-coated *S. pruinosus* rapidly decreased during storage at 24 °C and 90% RH. The authors attributed this behavior to the high rate of transpiration having significant differences in WL among variants, owing to the number of areolas [4]. Low temperatures and high RH are often the storage conditions to reduce the dehydration of cactus fruit, although the main constraint is the cold damage. Ali et al. [26] indicate that a WL of 27.8% after 28 days of storage of dragon fruit (Hylocereus polyrhizus Weber) at 10 °C and 80% of RH, while WL of fruit with CH and submicron CH dispersions varies from 15% to 18%. Nevertheless, pitaya (Stenocereus spp.) is more delicate and intricate to preserve than dragon fruit, in agreement with Armella et al. [1]. These authors observed a rapid WL (8%) after only 8 days at 7 °C and 85% RH.

There are significant differences (*p* < 0.05) among fruits with and without coating (control) at 10 °C and 80% RH. NCHH reduces to a lesser extent the weight of pitaya than the control and other treatments after 15 days of postharvest (Figure 1).

#### 2.2.2. Determination of Contact Angle on the Epicarp of Pitayas

Generally, the contact angle of the surface of plants varies from 45° and up to 150° and beyond [27]. According to Vogler [28], a hydrophilic surface presents a contact angle lower than 65°, while the opposite for hydrophobic ones (>65°). The epicuticular wax layers in plants are hydrophobic with an influence on the wettability of plant surfaces, while intracuticular waxes control the permeability to water [27]. The contact angle of the epicarp of pitaya (control) indicates hydrophilicity (61.25 ± 0.33°) and despite the use of a hydrophilic polysaccharide coating with weak water vapor barrier characteristics, all coatings maintained the hydrophilic nature of the fruit, although NCHH is able to modify the contact angle to slightly hydrophobic. Thus, NCHH improves the moisture barrier, thereby restricting the weight reduction during storage (Table 2).

The contact angle decreased in CH and NCHMG, 50.04 ± 0.48° and 50.01 ± 0.32°, respectively, owing to the higher hydrophilicity than epicarp in the control fruit. By contrast, CHH, that reduces the WL with respect to the control (0.382 ± 0.057 day^−1^), is not significantly different for the contact angle (61.79 ± 0.53°). The crosslinking with citric acid might explain this experimental evidence as it decreases the hydroxyl moieties of CH, thereby reducing hydrophilicity in agreement with Moller et al. [15]. Despite nonsignificant differences with the control, the type of coating explains the lowest rate (0.289 ± 0.069 day^−1^) and highest contact angle (76.47 ± 0.45°) for NCHH, but most importantly, the emulsion breakdown that releases the oil, thereby reducing water migration and WL.

#### 2.2.3. Determination of pH, Titratable Acidity (TA), and Total Soluble Solids (TSS)

**Figure 2:**
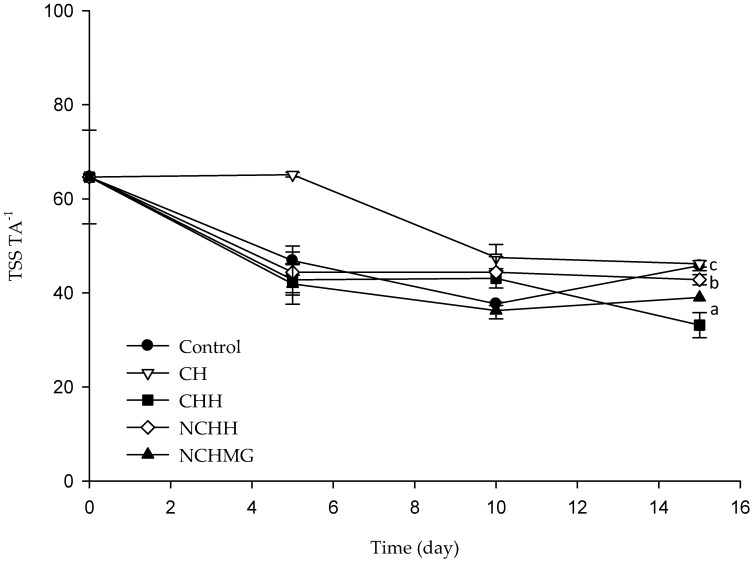
Ratio of Total Soluble Solids (TSS) and Titratable Acidity (TA) (TSS TA^−1^) of pitaya (*S. pruinosus*) during storage at 10 °C and relative humidity (RH) of 80% (See Table 2 for treatment identification). Data are the mean and their standard deviation (*n* = 9). Different letters among traces mean significant differences (*p* < 0.05).

The acidity in fruits due to the presence of organic acids, such as malic, ascorbic, citric, tartaric, and lactic and oxalic acids is not known for *S. pruinusus* [4]. In the present work, the TA is a percentage of malic acid, as the main organic acid related to *Hylocereus* [29], and renders no significance (*p* > 0.05) in samples during storage ranging from 0.16% to 0.30%. Additionally, the pH and TSS data remain without changes during storage from 5–5.9 and 10 and 12.5° Brix, which are in the range for *S. pruinusus*, in agreement with other reports [4] (Figure S3 shows pH, TSS, and TA data). On the other hand, the TSS TA^−1^ (Figure 2) rates concomitant to sweetness or acidity displays significance for coated fruits (*p* < 0.05), where the control and CH are the sweetest, while CHH and NCHMG have the most acid. Sample NCHH is among both groups of significance according to the Tukey test.

#### 2.2.4. Effect of Coatings on Color, Betalains, Phenolic Compounds, and Ascorbic Acid in Pitaya

**Figure 3:**
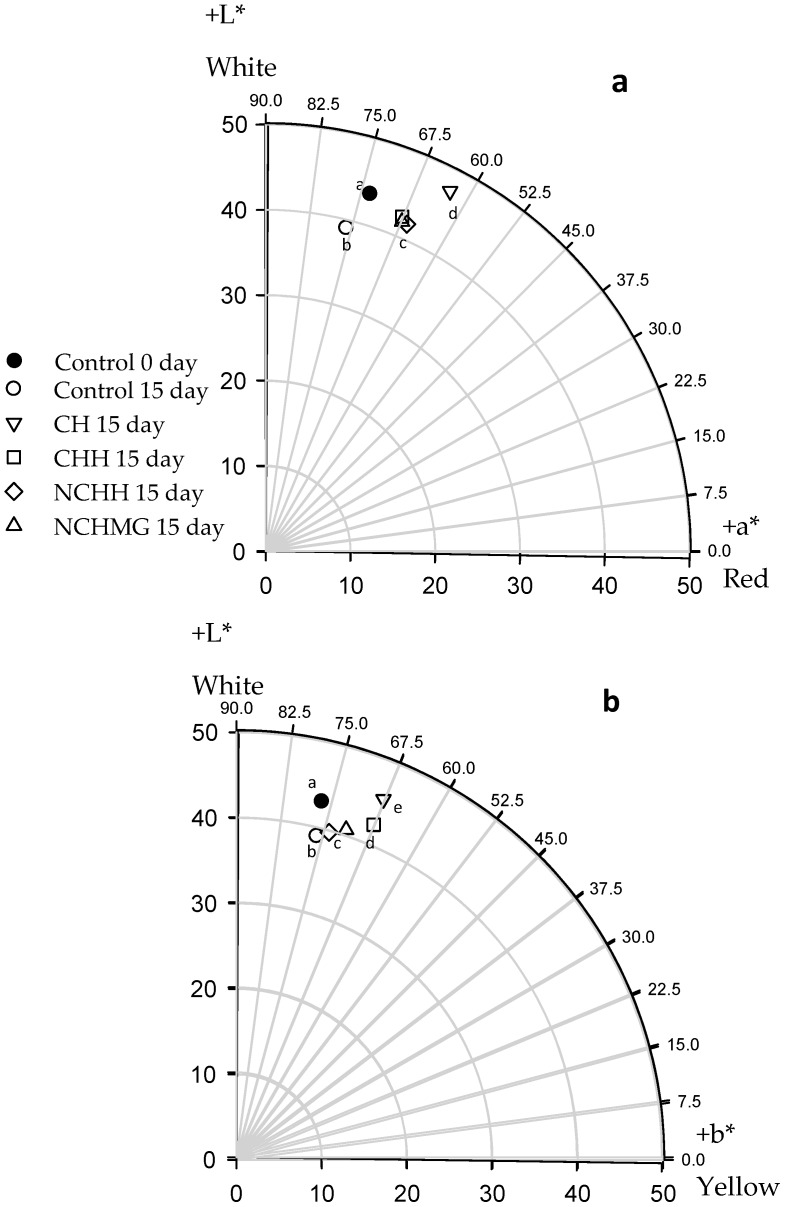
Color measurements on the epicarp of pitayas: (**a**) 0 day and (**b**) 15 days of storage at 10 °C and relative humidity (RH) of 80% (See Table 2 for treatment identification). Data are the mean (*n* = 27). Different letters among treatments mean significant differences (*p* < 0.05).

The CIELAB parameters are significantly different among treatments (*p* < 0.05) and, interestingly, all coated samples display increasing brightness (L*) compared to the control (Figure 3). Noteworthy, L* is one of the most critical parameters to describe the consumers appealing for the fruit. The experiment raises an interesting outcome considering that previous work has reported a negative impact of chitosan containing 1% or 2.5% acetic acid coatings on red prickly pear [13]. These authors describe remarkable changes in the total color because of the darkening and reduction of a*. In the present work, there are also significant differences among treatments for a* and b* (*p* < 0.05); CH treatment has the highest red coloring whereas the lowest is the control. The yellow coloring of fruit coated with CH and CHH displays significant differences compared to other coatings and the control (Figure 3). According to García-Cruz et al. [8], the changes in color are due to epicarp betalains in *S. pruinosus*, which corresponds to the sum of red/purple betacyanins and yellow betaxanthins, respectively [30].

**Figure 4:**
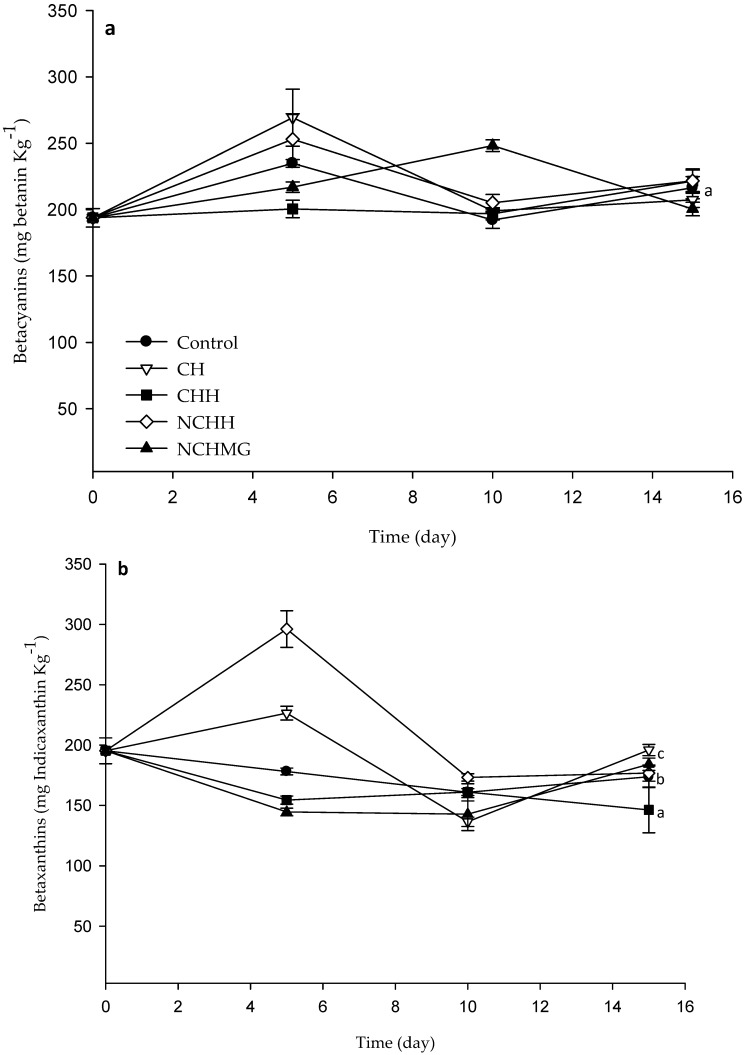

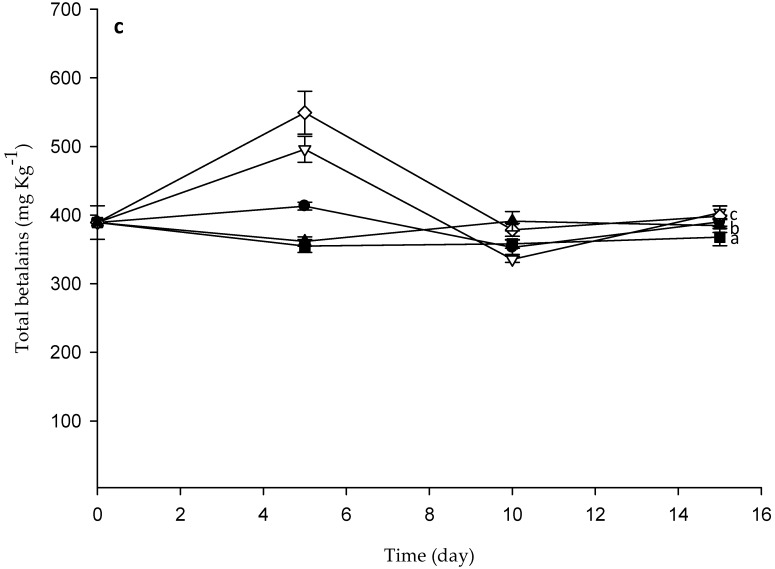
Determination of betacyanins, (**a**), betaxanthins (**b**), and total betalains (**c**) in the pulp of pitaya during storage at 10 °C and relative humidity (RH) of 80%. Data are the mean and their standard deviation (*n* = 9) (See Table 2 for treatment identification). Different letters among traces mean significant differences (*p* < 0.05).

Concomitantly, after 15 days of storage, the WI has no differences among treatments and only NCHMG increased WI, while the control significantly reduces. A similar pattern is observed for RI and YI, in which the coated fruits show significantly higher values than the control (Table 2). Clearly, the changes in the color of epicarp in the control relates to betalains’ degradation. In this regard, the radical scavenging activity of Neem and the acidic pH probably delays degradation of this pigment in fruit coated with the chitosan-based materials. On the other hand, Figure 4 shows betacyanin and betaxanthin concentrations in the pulp for control and coated samples, with significant differences (*p* < 0.5) during storage. The application of NCHH and CH on the pulps results in the highest concentration of these pigments, thereby preserving epicarp and pulp colors, thus enhancing human health. This result agrees with García-Cruz et al. [4], where the *S. pruinusus* storage for 6 days at 24 °C rendered non-significant changes in total betalain concentration. Additionally, there are other antioxidants, such as phenolic compounds and ascorbic acid, that might prevent pigments’ degradation. These compounds’ concentrations have no significant variations (*p* > 0.5) during storage (Figure S4 shows phenolic compounds and ascorbic acid data) and they are within the range of that by García-Cruz et al. [4] in pitaya at 24 °C for 6 day. Noteworthy, the variation of 0.10–0.17 g·kg^−1^ agrees with the results of Beltrán-Orozco et al. [6] in pitaya, *Stenocereus stellatus*.

#### 2.2.5. Pulp Firmness and Sensorial Analysis

The firmness of the pulp control and coated pitayas during storage showed no significant differences (Figure S5 shows the firmness of pulp of pitayas data). There is, however, a slight loss of firmness of the pulp, owing to senescence by degradation of the polysaccharides of the cell walls by enzymatic reactions, such as that associated to pectin methylesterase and polygalacturonase [31]. Sensory analysis for the fruit at the beginning and at the end of the essay concluded that NCHH has no effect on the appealing of the fruit. On the other hand, CH and NCHMG enhanced the general appearance, aroma, and consistency although there is a change in taste. CHH improves the appearance, but lowers the consistency of the pulp (Figures S6 and S7 show the sensory analysis results of control and coated fruits).

### 2.3. Fungal and Mesophilic Aerobic Bacterial Contamination of Samples Fruits’ Control and Coated Samples during Storage

**Figure 5:**
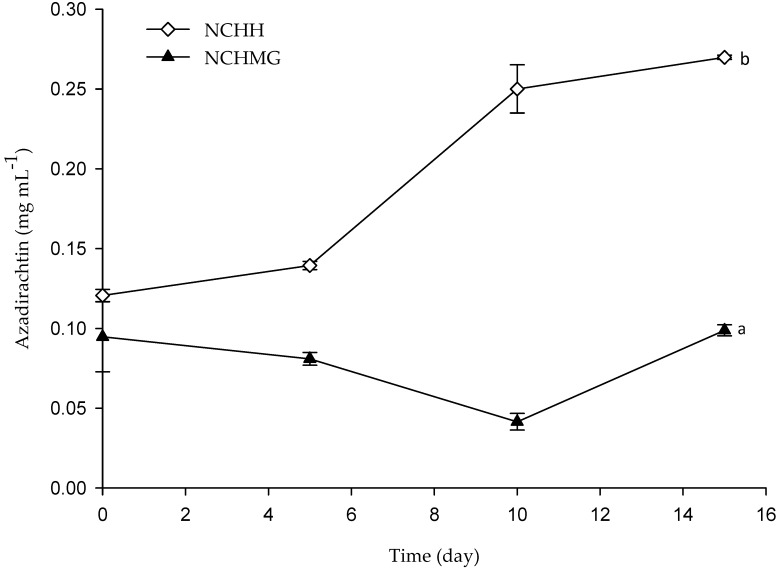
The release of azadirachtin of Neem during storage at 10 °C and relative humidity (RH) of 80%. (See Table 2 for treatment identification). Data are the mean and their standard deviation (*n* = 9). Different letters among traces mean significant differences (*p* < 0.05).

**Figure 6:**
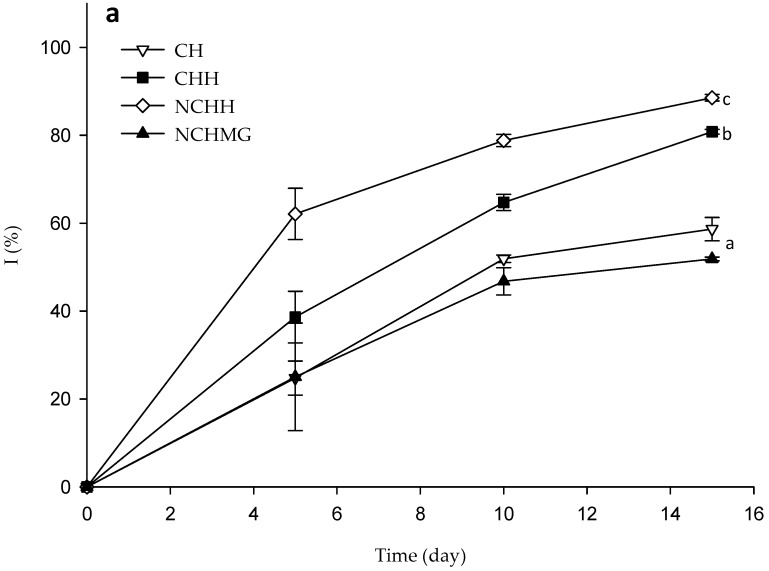

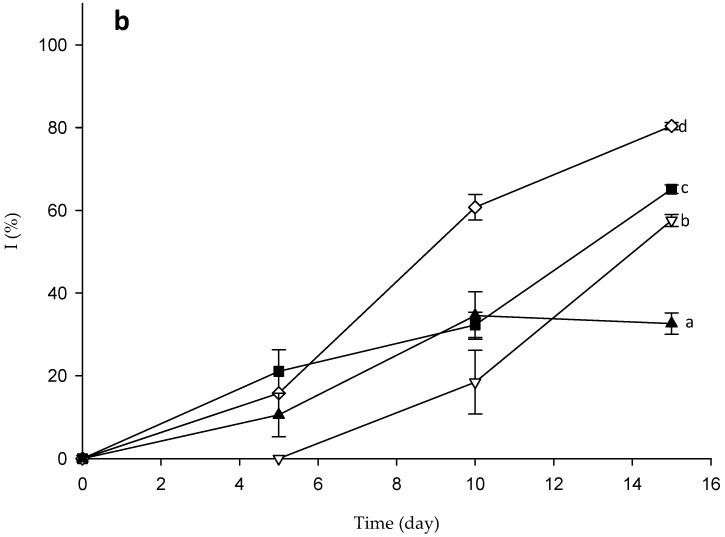
Fungal (**a**) and mesophilic aerobic bacterial (**b**) inhibitions on the epicarp of pitayas during storage at 10 °C and relative humidity (RH) of 80% for control and coated fruits (See Table 2 for treatment identification). Data are the mean and their standard deviation (*n* = 3). Different letters among traces mean significant differences (*p* < 0.05).

The coating application on the pitaya fruits restricts water loss, thus providing protection to the surface and maintaining the turgidity of the fruit. Besides, the appearance improves as the coating conveys brightness (Figure S8 shows photographs of fruits during storage). The NCHH coating has the highest fungal and mesophilic aerobic bacterial inhibition during storage with 96.2 ± 1.1% and 93.9 ± 1.1%, respectively, while CHH (78.0 ± 2.2% and 80.5 ± 1.4%) and CH (76.1 ± 3.8% and 88.3 ± 2.0%) present lesser antimicrobial activity. On the other hand, NCHMG coating displays moderate antimicrobial activity (51.8 ± 7.8% and 46.1 ± 3.6%) despite the presence of CH and Neem (Figure 5 and Figure S8). This poor result might be ascribed to MG, a poly (1–3) β-d-galactose with (1–6) branching of l-arabinose, l-rhamnose, β-d-glucuronate, and 4-*O*-methyl-β-d-glucuronate, which sustains fungal growth [24]. Noteworthy, the control media has the exact acetic acid concentrations to that of the coating formulations to assess the antimicrobial effects (Figure 6).

The isolated fungal colonies from epicarps of the control and NCHMG-coated pitaya present a green color with 25 to 30 mm colony diameters. The hyphae septate and conidiophores are scarce and geniculate, and the septa distribute irregularly transverse. The conidia are obclavate, large, and with a muriform shape (Figure S9 shows photographs of light microscopy of fungal isolate at 100-magnification). According to these morphological characteristics, this fungus is *Alternaria*, owing to the conidia chains, whereas *Ulocladium* usually assembles as single or short chains [32]. 

### 2.4. Release of Neem from Coatings

Neem extraction from the Neem seeds contains ca 40% of the triterpenoids-rich oil. Therein, salannin and azadirachtin are the most abundant polar limonoids. The Azadirachtin has antimicrobial and inhibitory activity on insect growth. Several reports indicate moulting defects and sterility due to the blockade on microtubule formation in dividing cells. The mode of action of azadirachtin in the protozoa, *Tetrahymena thermophilae*, relies on cell proliferation and interference to RNA synthesis [33]. In addition to these applications, Neem extracts from leaves and kernels are residue-free and safe for human consumption, which stands out against synthetic fungicides. Additionally, Raizada et al. [18] claimed that the azadirachtin is a non-mutagenic compound without mammalian toxicity and without signs of toxicity, mortality, changes in tissue weight, pathology, and serum and blood parameters after in vivo toxicological trials in rats for 90 days. The determination of azadirachtin by UPC2 evidences the release of the oil from the emulsions (Figure 5) as the concentration significantly increases during storage of NCHH-coated pitayas. Noteworthy, NCHMG coating shows no release of the oil according to the stability of the emulsion due to the pH on the pericarp (5.2 ± 0.1) and storage conditions in terms of the temperature and RH. A different azadirachtin concentration at the initial time is due to the low stability of the NCHH coating as it releases N after 45 min at room temperature (see Section 2.1).

### 2.5. Changes in Morphology of the Control and Coated Pitaya Epicarps by SEM

**Figure 7:**
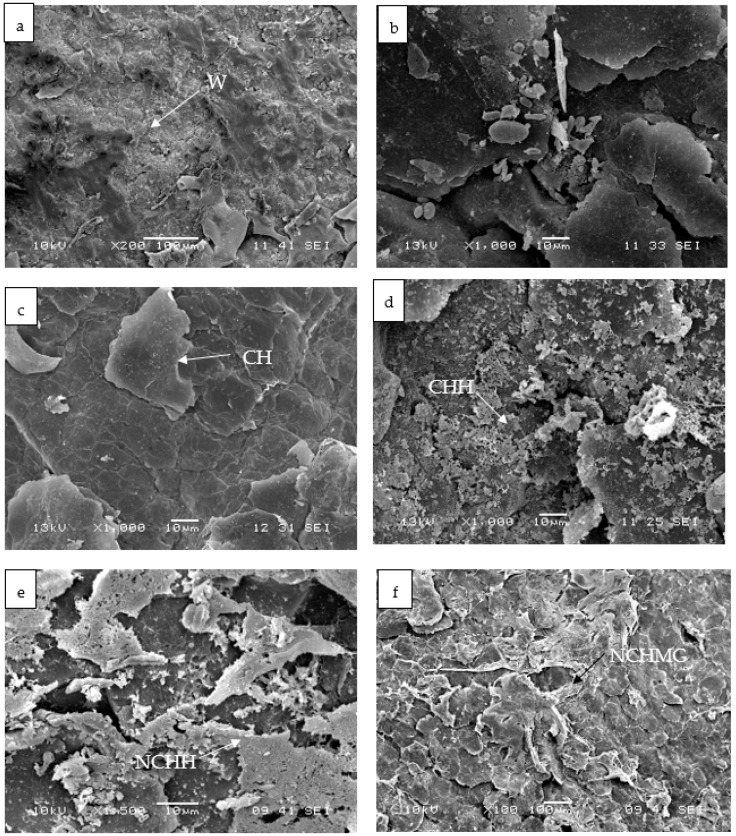

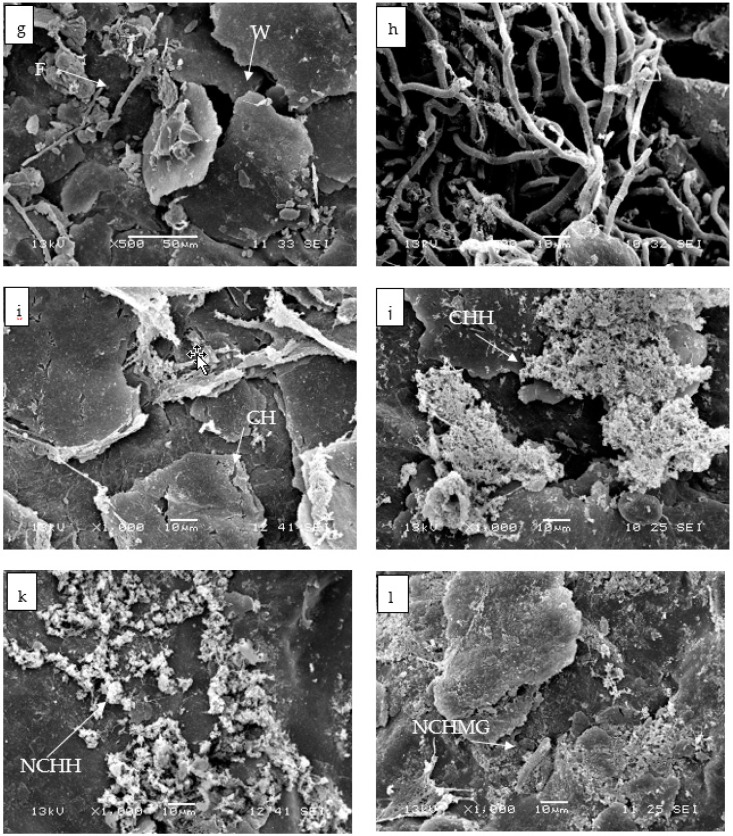
SE micrographs of coatings on pitayas at the initial time: Control (**a**), *Alternaria* inoculated (**b**), CH (**c**), CHH (**d**), NCHH (**e**), and NCHMG (**f**). At 15 d of storage: Control (**g**), *Alternaria* inoculated (**h**), CH (**i**), CHH (**j**), NCHH (**k**), and NCHMG (**l**). (See Table 2 for treatment identification). W is a natural wax, and F is fungi (three preparations per treatment with 10 observations in SEM).

The areola of pitaya is a structure formed by a series of radial and central spines that capture water from the environment [34]. These areolas are easily withdrawn after harvest, causing a higher migration of water, which leads to a rapid WL. According to García-Cruz et al. [4], *S. pruınosus* presents 24 to 28 areolas on average, and the number of areolas is proportional to WL. The areola observation by SEM in the experimental units of this work, shown in Figure S10, presents two types of spines, central and radial, as well as the wax (Figure 7a), which is a natural coating in the pitaya [34]. Holloway and Jeffree [27] suggests that a surface contact angle lower than 90°, similar to this work (Table 2), is a result of the incomplete covering of wax on the epicarp, resulting in roughness and interstices (Figure 7). Nonetheless, the epicarp possesses corrugations and trichomes that might enhance the wetting. Noteworthy, the harvested pitayas are in wooden boxes, where spines perforate the epicarp of proximal fruit, producing damages and accelerating water losses [10]. The SEM in Figure S10 illustrates the damage on the epicarp.

The CH coating forms a smooth and homogeneous surface, despite the irregular morphology of the epicarp (Figure 7b), whereas those for CHH and NCHH are heterogeneous (Figure 7c,d). Similarly, NCHMG produces a network structure onto the epicarp and agglomerations in other areas due to the hydrophilicity of MG (Figure 7e).

Figure 7 shows the SE micrographs at the end of the bioassay, where the control (Figure 7f) sample retains the characteristically natural wax, but evidences the growth of microorganisms in agreement with the microbial counts. Alternaria is inoculated on pitaya epicarps to distinguish the fungal structures (Figure 7b), in addition to septate hyphae as well as conidia chain observations (Figure 7h). By the end of the bioassay, CH (Figure 7i) remains on the epicarp of the cactus, but CHH (Figure 7j), NCHH (Figure 7k), and NCHMG (Figure 7l) have agglomerations. There is an incomplete covering of wax of the epicarps that explains this roughness and interstices (Figure 7). Nonetheless, the epicarp has other surface features, such as corrugations or trichomes, that might enhance the wetting and coating interaction. Moreover, the coating has no dramatic changes on the ripening of these non-climacteric fruits or change of flavor. Nevertheless, despite the nutraceutical potential of this fruit, there are no reports on the characterization of the ripening and identification of postharvest diseases.

## 3. Materials and Methods

### 3.1. Materials

J.T. Baker (Ecatepec State of Mexico, Mexico) supplied the citric acid monohydrate, sodium phosphate monobasic, d-sorbitol, sodium hydroxide, and petroleum ether. Hydroxypropylmethylcellulose (H) was a kind gift from Derivados Macroquímicos (Ecatepec Morelos, Mexico). Neem Mex (Quebrantadero Morelos, Mexico) supplied Neem oil. Sigma-Aldrich, St. Louis, MO, USA, supplied 2,2-diphenyl-1-picrylhydrazyl (DPPH), poly(vinyl alcohol) (PVA), azadirachtin as external standard, trichloroacetic acid, and 1,10-phenanthroline, gallic acid. Acetic acid, ethanol, methanol, phosphoric acid, ferric chloride, calcium carbonate were purchased from Reactivos Quimica Meyer (Mexico City, Mexico). Farmacias Paris (Mexico City, Mexico) provided ascorbic acid. Folin-Ciocalteu, Tween 20, Tween 80 were purchased from Hycel (Mexico City, Mexico), All reagents were analytical reagent grade. Exudate extracts from Mesquite (*Prosopis*) trees in San Luis Potosi (Mexico) provided the Mesquite gum (MG) after purification according to methodology reported by Vernon-Carter, Beristain, and Pedroza [24].

### 3.2. Preparation and Characterization of CH and CHH

Thermochemical deacetylation of chitin from shrimp wastes using *Lactobacillus brevis* as the starter following an earlier report [35] produced CH. DA was the result of proton nuclear magnetic resonance (^1^H-NMR) spectroscopy analyses in a Bruker (AC 200) at 200 MHz using D_2_O with 10% HCl with 3-(trimethylsilyl) propionic acid as an internal reference [35]. The Mark-Houwink-Kuhn-Sakurada equation allowed the CH viscosimetric average molecular weight (Mv) by capillary viscometer using acetate buffer solution.

In a typical procedure for CHH preparation, CH (5 g·L^−1^) is dissolved in 0.1 M of acetic acid. 15 g of H was dissolved in 644 mL of distilled water and stirred with mechanical agitation for 24 h, afterwards 322 mL of ethanol and 3.2 mL of polyethylene glycol were added. H and CH solutions were mixed in a ratio of 1:1 (vol:vol). Citric acid (50 g·L^−1^), NaH_2_PO_4_ (37.5 g·L^−1^) and d-sorbitol (10 g·L^−1^) were incorporated as crosslinker, catalyst, and plasticizer, respectively. The mixture was stirred with continuos mechanical agitation until complete dissolution. Then, the mixture was placed under vacuum at 70 °C for 3 min [23]. The CHH solution was dried at 60 °C for 6 h. The resulting films were purified with methanol anhydrous in a ratio of 1:10 (wt:vol) for 24 h, dried, and dissolved in HCl/D_2_O for ^1^H NMR characterization. The hydroxypropylmethylcellulose cross-linked to CH molar ratio for CHH was calculated from integration of the assigned signal of the massive considering the acetyl of CHH to the massive ratio of the acetyl of native CH signals in the ^1^H NMR.

### 3.3. Characterization of Neem: Determination of Azadirachtin Concentration and Radical Scavenging Activity

Azadirachtin in Neem was determined by UltraPerformance Convergence Chromatography (UPC2) (Waters ACQUITY, Milford MA, USA) equipped with a photodiode array detection and a C-18 column (Waters 1.0 × 100 mm, 1.7 μm) using supercritical CO_2_ and methanol as the mobile phase (Flow rate = 1.5 mL·min^−1^), convergence pressure of 1500 psi, and column temperature of 30 °C. The sample was dissolved in methanol: Petroleum ether of 5 °C [36]. Inhibition of radical scavenging activity (RSA) was determined spectrophotometrically with Neem concentrations from 5 to 25 µg·mL^−1^ with freshly prepared DPPH (0.1 mM/methanol) (Sigma-Aldrich, St. Louis, MO, USA). Test tubes were stirred in a vortex followed by incubation at 30 °C for 30 min in darkness. Absorbance at 517 nm was measured in a Genesys UV-visible spectrophotometer (Thermo Fisher Scientific, Waltham, MA, USA) by triplicate. RSA (%) was determined from Equation (1), considering the absorbance of ethanol as the control (A_0_) and the sample (A_X_):

(1) $$RSA\;(\%)=\left(\left(A_{0}-A_{x}\right)/A_{0}\right)\times 100$$

Experimental data were fitted to Probit statistical analysis in the NCSS^®^ program [37]. The IC_50_ value was the concentration of N that inhibited 50% of DPPH [38].

### 3.4. Emulsions Formulation and Characterization of Chitosan-Based Coatings with Neem Oil

CH solution (5 g·L^−1^) in acetic acid (0.1 M) was the coating CH. CHH coating was prepared as described in Section 3.2 and applied on the fruit without purification. For the preparation of Neem incorporated coatings, emulsions of oil in water with a ratio of dispersed phase and continuous phase of 1:9. The mixture of N (16 g·L^−1^) and mineral oil (84 g·L^−1^) in a ratio of 1:5.25 (wt:wt) was the dispersed phase that was added dropwise into the aqueous solution of MG (10 g·L^−1^), homogenized for 10 min at 40 Hz (Hielscher up 400S, Teltow, Germany), and subsequently added dropwise to CH solution (5 g·L^−1^ in acetic acid (0.1 M)). CH and MG were the continuous phase in a ratio of 1:20 (wt:wt). pH was adjusted to 4.5 with NaOH (0.1 N) to obtain NCHMG. For the other emulsion coating, the dispersed phase consisted of tween 20 (0.9 g·L^−1^), tween 80 (0.6 g·L^−1^), PVA (10 g·L^−1^), Neem (16 g·L^−1^), and mineral oil (72.5 g·L^−1^) were added dropwise to the continuous phase of CHH. The mixture was stirred until an oil-in-water emulsion was homogenized with an Ultra Turrax Ika 25 (Janke and Kunkel GmbH and Co., Staufen, Germany) at 10,000 rpm for 10 min to obtain NCHH. The ratio of dispersed phase and continuous phase was 1:9.

### 3.5. Droplet Size and Zeta Potential Determinations

Droplet size distribution of the emulsions was carried out by the laser diffraction method in a Master sizer 2000 (Malvern Instruments, Malvern, Worcestershire, UK). Average Sauter diameter (D_3.2_) (Equation (2)) and polydispersity through the dispersion index (Span) measurements have the assessment of the variations in the droplet sizes for a given emulsion (Equation (3)).

(2) $$D_{3.2}=\sum n_{i}d_{i}^{3}/\sum n_{i}d_{i}^{2}$$

where *n_i_* is the number of particles with diameter i (*d_i_*).

(3) Span=(d(90)−d(10))/d(50)

where *d*(90), *d*(50), and *d*(10) represent the diameters to the cumulative distribution at 90%, 50%, and 10%, respectively.

Zeta potentials (ζ) of the CH, hydroxypropylmethylcellulose, MG, and emulsions were determined in a Zetasizer Nano ZS (Malvern Instruments Ltd., Malvern, Worcestershire, UK). pH was adjusted in a pHmeter (pH210 Hanna Instruments, Woonsocket, RI, USA) equipped with an electrode (HI 1332 Hanna Instruments, Woonsocket, RI, USA). Emulsion stability was determined by measuring the upper oil-rich layer formation after leaving it to stand in 30 mL of each emulsion in conical graduated screw cap tubes and stored upright at 25 ± 1 °C. Formation of upper cream-like layers was observed every 15 min for the first 9 h, and then every 24 h for two weeks and then once per week.

### 3.6. Application of Coatings on Pitaya Fruit

Pitayas (*S. pruinosus*) were harvested in May 2015 from an orchard in Mixteca region in the state of Puebla, Mexico (17°48′00″ N, 97°46′00″ O) and were selected at the commercially mature stage, identified with easily detached spines and shiny skin. After the harvest, the spines were removed from the fruit, and subsequently, the fruit with red flesh was chosen because it contained the highest amount of betalains [4]. Fruit samples were sorted for defects (38 kg) and randomly distributed into five groups, which corresponded to 15 experimental units for each treatment. Units were placed in clamshell containers with three fruit each (ca. 0.5 kg), as control; CH; CHH; NCHMG and NCHH. Fruit was immersed for 1 min in the coating solution followed by drying at room temperature for 1 h and stored at 10 ± 2 °C and 80 ± 3% relative humidity (RH). The trial was repeated thrice with three different pitaya batches.

### 3.7. Fruit Quality Evaluation

All experimental units were weighed (Ohaus, Parsippany-Troy Hills, NJ, USA) every 24 h during storage. Percentage of weight loss (% WL) was calculated according to the initial weight. WL data was fitted to a linear model, the level of significance of each independent variable and squared multiple correlation coefficients (R^2^) were estimated using the regression software, STATISTICA (StatSoft, Inc. Tulsa, OK, USA). A portable refractometer (Atago Co., Tokyo Japan) determined the total soluble solids (TSS) in the extracted juices in Brix. Volumetric titration with NaOH (0.01 N) (AOAC method) determines the titratable acidity (TA) in the juice of fruit as a percentage of malic acid as the dominant organic acid in the fruit. pH in juice fruit was conducted with a pHmeter (pH210 Hanna Instruments, Woonsocket, RI, USA) equipped with an electrode (HI 1332 Hanna Instruments, Woonsocket, Rhode Island, USA). Chromameter CR200 colorimeter (Minolta, Osaka, Japan) measured the color change of the epicarp in three equidistant equatorial and longitudinal parts of each fruit, and was expressed as L*, a* and b* according to the International Commission on Illumination (CIE). A white reference standard was used to calibrate the instrument. Whiteness (WI), yellowness (YI), and redness (RI) indexes were calculated with Equations (4)–(6), respectively [39].

(4) $$WI={\left(\left(100-L^{*2}\right)+a^{*2}+b^{*2}\right)}^{1/2}$$

(5) $$YI=142.86b^{*}/L^{*}$$

(6) $$RI=a^{*}/b^{*}$$

Firmness was measured in the flesh taking two random points of the equatorial region of each peeled fruit and expressed in N using a texturometer (Brookfield CT3, Middleboro, MA, USA) with a 4 mm diameter probe and 5 mm penetration distance with 1 mm·s^−1^ speed [13]. The determinations were the mean of 4 repetitions.

### 3.8. Determination of Total Betalains, Phenolic Compounds, and Ascorbic Acid (ASA) in the Flesh of Pitaya

The flesh of pitaya (2 g) was soaked with 20 mL of methanol (80% *v*/*v*) and sonicated for 10 min in a bath (Branson, MO, USA). The methanolic extract was stirred for 20 min at 25 °C and centrifuged at 2200× *g* for 10 min (Thermo Scientific, Legend XTR, Waltham, MA, USA). The pellet was subjected to a second extraction and the supernatants were collected and filtered (Columbia Filter, Tlalnepantla, Mexico Mexico). The filtrate was concentrated at 40 °C in a rotary evaporator (Büchi Labortechnik AG, Postfach, Flawil Switzerland), and re-suspended in 10 mL of methanol (80%) [4]. The concentrations of betacyanins and betalains were determined in the methanolic extract by spectrophotometry (Equation (7)). The total content of betalains, as the sum of betacyanins and betaxanthins, was expressed as mg per kg of fresh pitaya weight.

(7) $$B=\left(A\times D_{f}\times W\times V\right)/\varepsilon \times P\times l$$

where *B* is the content of betacyanins o betaxanthins, *A* is the absorbance (538 nm and 483 nm for betacyanins and betaxanthins, respectively), *D_f_* is the dilution factor, W is the molecular weight (550 g·mol^−1^ and 308 g·mol^−1^ for betanin and indicaxanthin, respectively), *ε* is the molar extinction coefficient (60,000 L·mol^−1^·cm^−1^ and 48,000 L·mol^−1^·cm^−1^ for betanin and indicaxanthin, respectively), *P* is the mass of the sample (g), and *l* is the length of the cell (1 cm) [40]. 

The phenolic compounds were determined from the reaction of 0.1 mL of the methanolic extracts and 0.25 mL of Folin-Ciocalteu reagent diluted with distilled water in the ratio of 1:1 (vol:vol). After 6 min, 1.25 mL of a Na_2_CO_3_ solution (190 g·L^−1^) was added and the volume adjusted to 3 mL with distilled water. The mixture was left in darkness at room temperature for 90 min prior to measurement at 760 nm. The concentration of phenolic compounds was estimated from the calibration curve using gallic acid as the standard and expressed as (mg·kg^−1^) [8].

For the ascorbic acid (AsA) determination, 2 g of flesh was macerated with 5 mL of trichloroacetic acid (5% *w*/*v*), sonicated for 10 min, followed by agitation for 20 min and centrifugation (2200× *g* for 10 min). To 1 mL of the supernatant, 0.5 mL of trichloroacetic acid solution, 1 mL of ethanol, 0.5 mL of phosphoric acid (0.4% *w*/*v*), 1 mL of 1,10-phenanthroline (0.5% *w*/*v*), and 0.5 mL of FeCl_3_ solution (0.03% *w*/*v*) were added. The mixture of reaction was incubated for 1 h to measure the absorbance at 534 nm (Genesys 6, Thermo Scientific, Waltham, MA, USA). The content was determined by a calibration curve of AsA standard and expressed as g·kg^−1^ [29].

### 3.9. Sensory Evaluation of Pitaya Treated with Chitosan-Based Coatings

The sensory evaluation was performed with a panel of 50 consumers (non-trained jury). The panelists rated the flesh on their appearance, aroma, flavor, and consistency with a 7-point hedonic scale from 1 extremely dislike to 7 extremely like. Ten g of each sample were served into cups coded with random numbers; drinking water and crackers were provided to the panelists. 

### 3.10. Determination of Contact Angle on Coated Pitaya

The contact angle was determined by the sessile drop method, placing a droplet of deionized water (2 μL) on different points of each epicarp surface in triplicate for coated and control pitayas. Images were taken with a side-illumination horizontal light microscope Intel Qx3 (Intel Corporation, Santa Clara, CA, USA) and processed by image analysis (ImageJ version 1.41o software, NIH, Bethesda, MD, USA).

### 3.11. Enumeration of Fungi and Mesophilic Aerobic Bacteria of Epicarps of Coated Pitaya

The coated and control epicarps (10 g) were homogenized in 90 mL of isotonic saline solution 0.9% NaCl (*w*/*v*). Serial decimal dilutions were prepared and transferred to Petri dishes with potato dextrose agar acidified to pH 3.5 with tartaric acid (1.4% *w*/*v*) or standard methods agar for fungal and total mesophilic aerobic plate counts, respectively. Inhibition percentage (*I*%) considered the fungal and bacteria counts in the control and each chitosan-based coating (Equation (8)). Acetic acid at 0.1 M, 0.05 M, 0.044 M, and 0.044 M were added to the culture media considering the controls of CH, CHH, NCHH, and NCHMG, respectively, to distinguish their antimicrobial activity from that of the chitosan-based coatings.

(8) $$I\%=\frac{CFUmL_{control}^{-1}-CFUmL_{coating}^{-1}}{CFUmL_{control}^{-1}}\times 100,$$

Fungal colonies from the inoculation of serial dilutions in acidified potato dextrose agar were isolated by the streaking method. Microscopic morphology was observed by methylene blue staining in a light microscope (Carl Zeiss, Oberkochen, Germany).

### 3.12. Azadirachtin of N Release from the Emulsions in Stored Experimental Units

Measurement of azadirachtin concentration determined the release of the oil from emulsions. For this purpose, the coated fruit was washed with 20 mL methanol and the methanolic concentrate was obtained by evaporation at 30 °C and injected in an UPC^2^ following the procedure described in Section 2.2.

### 3.13. Scanning Electron (SE) Microscopy Analysis of Pitaya Epicarp

Pitaya epicarp for each coating and control were cut into 4 mm × 10 mm samples and immersed in 5% (*v*/*v*) glutaraldehyde for 24 h. Then, samples were fixed with OsO_4_ 1% (*w*/*v*) for 2 h, dehydrated in a graded alcohol series, and covered with carbon and gold before examination in a JEOL JSM-5900 (LV, Tokyo, Japan) microscope [41]. Fresh fruit was inoculated with spore suspension of 1 × 10^4^ spores·mL^−1^ of *Alternaria* sp. (Culture Collection of Centro de Biotecnología Genómica of Instituto Politécnico Nacional, Reynosa Tamaulipas, Mexico) by a puncture and stored at 10 °C and 80% of RH for 9 days. Samples were taken and prepared for SEM analysis as described above.

### 3.14. Statistical Analysis

A completely randomized design was used for the experiments. All experimental data were subjected to analysis of variance (ANOVA) and multiple means comparison of Fisher’s LSD test (*p* < 0.05). Except for the sensory evaluation data, the remaining were analyzed by the non-parametric approach of Friedman test (*p* < 0.05). The statistical analyses were performed using the statistical software NCSS-PASS-GESS [37].

## 4. Conclusions

The application of the NCHH coating to pitaya stored at 10 °C in high RH was delayed the senescence the best, thus preserving the fruit color for up to 15 days. The experimental evidence indicates the release of azadirachtin from N significantly decreased the WL and fungal contamination. The chitosan-based coating with Neem extended the pitaya shelf life for up to 15 days, allowing the exportation of this fruit to international markets.

## Figures and Tables

**Table 1:** Average Sauter diameter (D_3.2_), dispersion index (Span), and zeta potential (ζ) of biopolymers and emulsions.

Biopolymer/Emulsion	pH	D_3,2_ (μm)	Span	ζ (mV)
CH	3.03 ± 0.02	ND	ND	38.90 ± 0.02 ^e^
CH	4.50 ± 0.01	ND	ND	32.40 ± 0.07 ^d^
Hydroxypropylmethylcellulose (H)	3.05 ± 0.03	ND	ND	−0.04 ± 0.02 ^c^
Hydroxypropylmethylcellulose (H)	4.52 ± 0.01	ND	ND	0.01 ± 0.001 ^c^
Mesquite gum (MG)	3.04 ± 0.04	ND	ND	−7.20 ± 0.07 ^b^
Mesquite gum (MG)	4.53 ± 0.02	ND	ND	−11.50 ± 0.03 ^a^
Emulsion of Neem in CH cross-linked to hydroxypropylmethylcellulose solution (NCHH)	3.01 ± 0.01	451.22 ± 1.03 ^b^	0.78 ± 0.01 ^a^	20.26 ± 0.91 ^b^
Emulsion of Neem in CH with added Mesquite gum (NCHMG)	4.51 ± 0.01	1.87 ± 0.87 ^a^	2.53 ± 0.41 ^b^	−15.30 ± 1.11 ^a^

Data are the mean and their standard deviation (*n* = 9). Different letters in the same column mean significant difference at *p* < 0.05. ND not determined.

**Table 2:** Weight loss (WL) rate, contact angle, whiteness index (WI), redness index (RI), and yellowness index (YI) of epicarp of control and coated pitayas during storage at 10 °C with relative humidity (RH) of 80%.

Treatment	Contact Angle (°)	WL Rate			Color Index			
		day^−1^	Significance Level	R^2^	Time (d)	WI	RI	YI
Control	61.25 ± 0.33 ^b^	0.42 ± 0.03	0.0001	0.988	0	60.53 ± 0.63 ^a^	1.35 ± 0.11 ^c^	33.83 ± 2.05 ^a^
					15	60.34 ± 0.65 ^a^	0.56 ± 0.05 ^a^	41.09 ± 1.98 ^b^
Chitosan (CH)	50.04 ± 0.48 ^a^	0.55 ± 0.03	0.0001	0.997	15	66.57 ± 0.63 ^c^	1.57 ± 0.08 ^d^	42.82 ± 1.87 ^b^
Chitosan cross-linked to hydroxypropyl-methylcellulose solution (CHH)	61.79 ± 0.53 ^b^	0.38 ± 0.06	0.0001	0.963	15	66.88 ± 0.52 ^c^	1.55 ± 0.06 ^d^	46.95 ± 1.45 ^c^
Emulsion of Neem oil in chitosan cross-linked to hydroxypropyl-methylcellulose solution (NCHH)	76.47 ± 0.45 ^c^	0.29 ± 0.07	0.0001	0.991	15	65.80 ± 0.37 ^c^	1.54 ± 0.10 ^d^	46.38 ± 1.84 ^c^
Emulsion of Neem oil in chitosan with added Mesquite gum (NCHMG)	50.01 ± 0.32 ^a^	0.58 ± 0.04	0.0001	0.969	15	64.19 ± 0.48 ^b^	1.25 ± 0.12 ^b^	48.95 ± 2.05 ^c^

Data are the mean and their standard deviation (*n* = 3 for WL and contact angle, *n* = 27 for color index determination). Different letters in a column are significantly different (*p* < 0.05).

## Supplementary Materials

Supplementary materials can be available online.
