# Supplementary material for: Cross-Linking Chitosan into Hydroxypropylmethylcellulose for the Preparation of Neem Oil Coating for Postharvest Storage of Pitaya (Stenocereus pruinosus)
Source: Molecules. 2019 Jan 9;24(2):219. doi: 10.3390/molecules24020219 (PMC6358988; doi:10.3390/molecules24020219)
Supplement: Supplementary file 1 [file molecules-24-00219-s001.pdf]

molecules

Supplementary Materials: Cross-linking Chitosan into Hydroxypropylmethylcellulose for the Preparation of Neem oil Coating for Postharvest Storage of Pitaya (*Stenocereus pruinosus*)

Carmen G. Hernández-Valencia, Angélica Román-Guerrero, Ángeles Aguilar-Santamaría, Luis Cira, Keiko Shirai

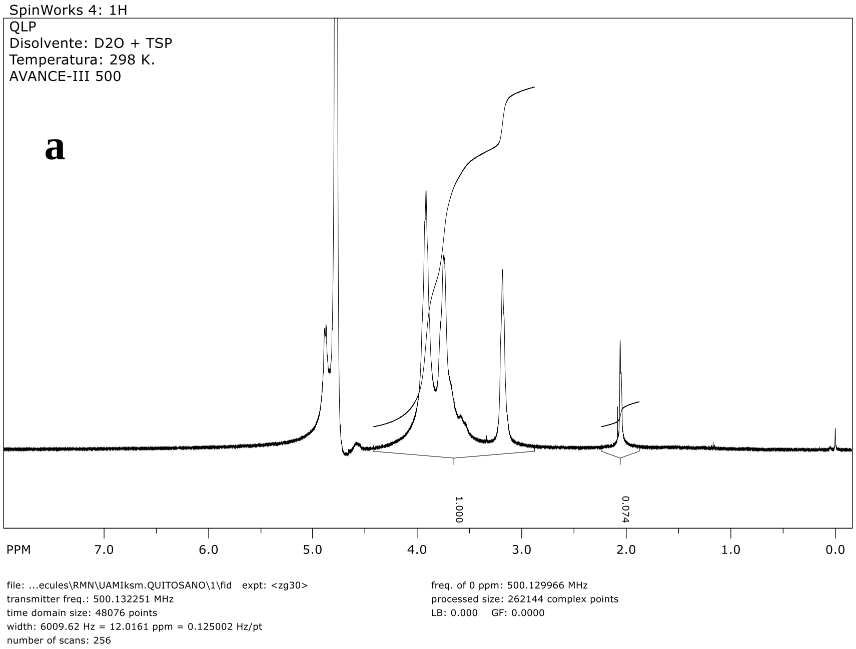

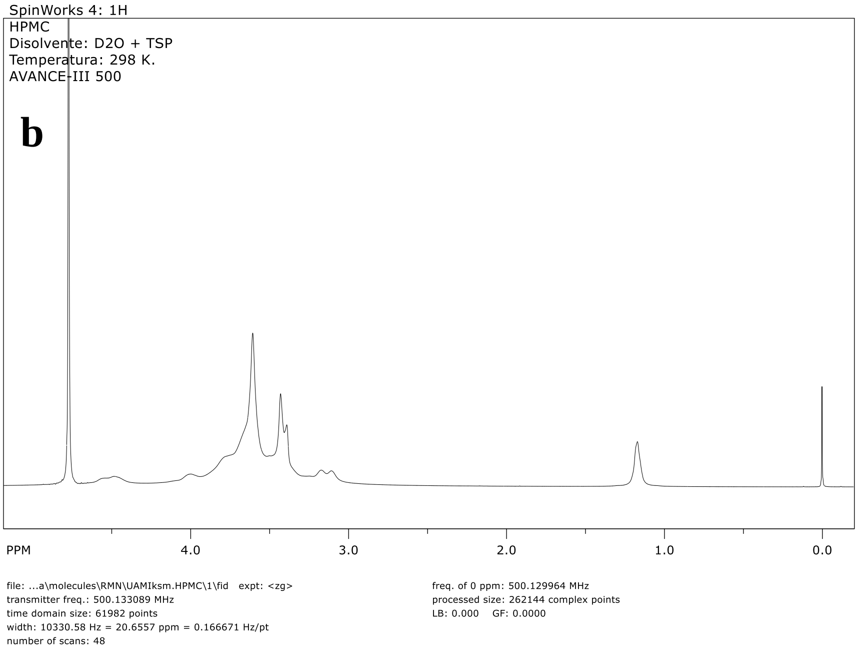

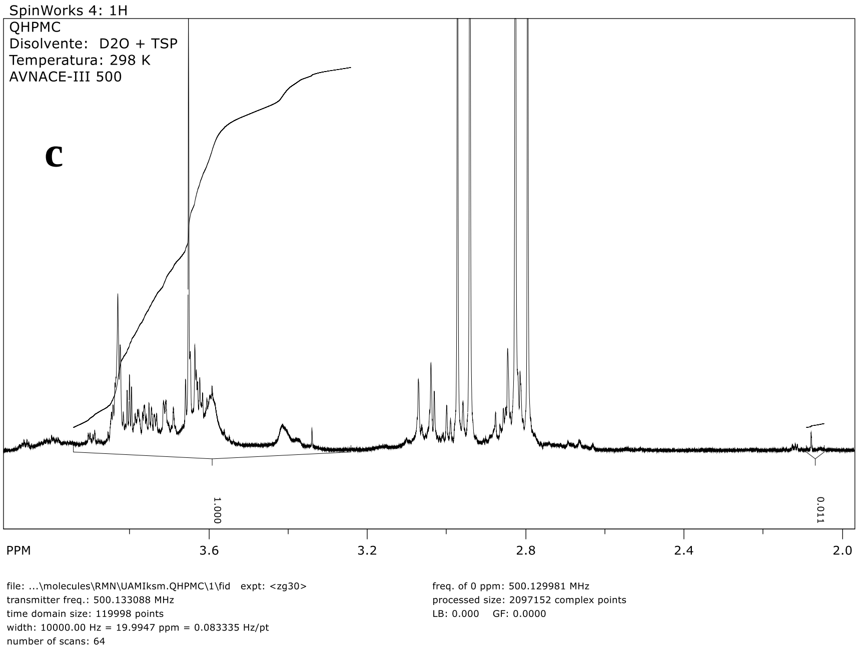

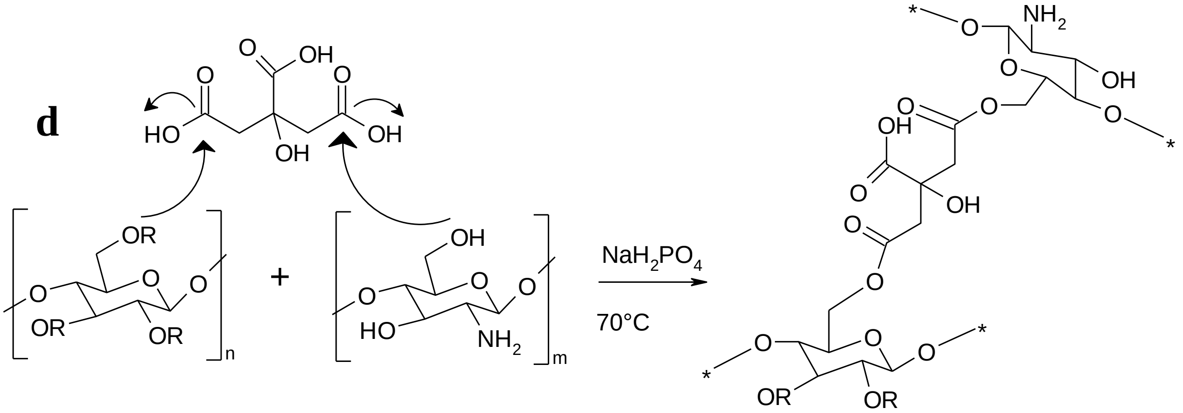

CH

CHH

Figure 1. Spectra of (a) CH, (b) hydroxypropylmethylcellulose (H) and CHH (c) obtained by ^{1}H-NMR; (d) scheme reaction of the cross-linking reaction between CH and hydroxypropylmethylcellulose with citric acid as cross-linking agent to a possible molecular structure in product.

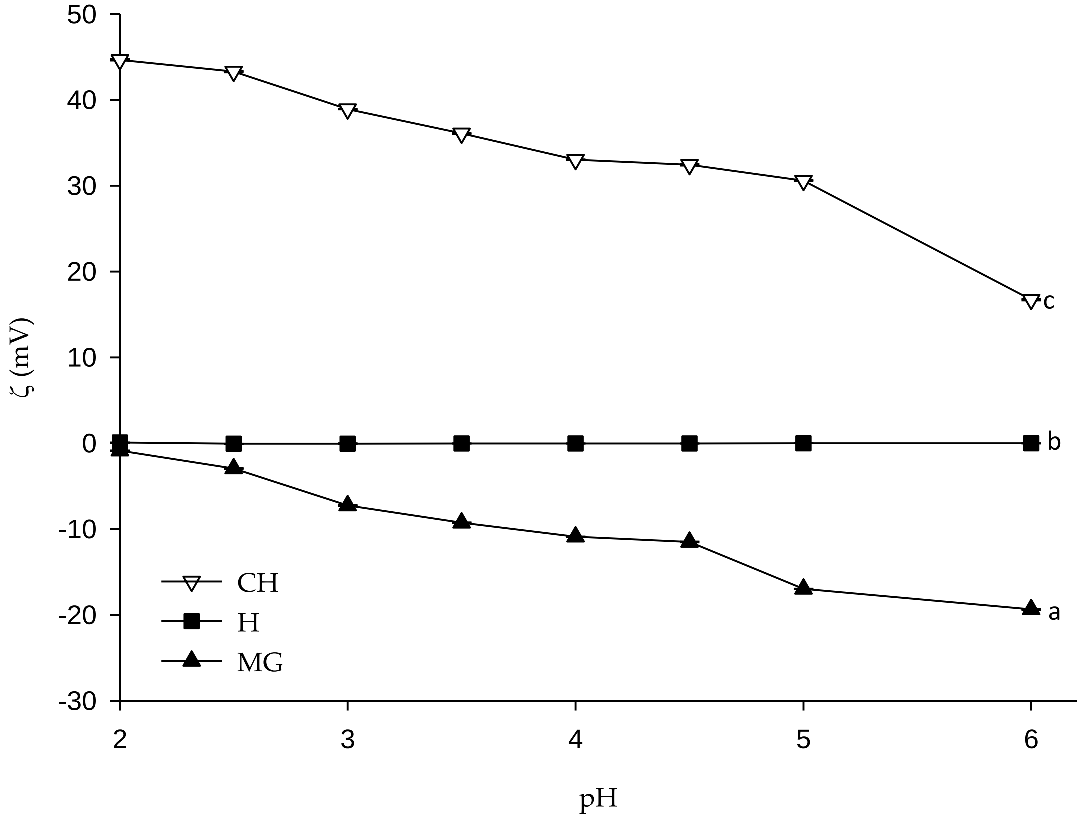

Figure 2. ζ Potential for CH, hydroxypropylmethylcellulose (H) and MG (Mesquite gum). Data are the mean and their standard deviation (n=100). Different letters among traces mean significant differences ($p < 0.05$).

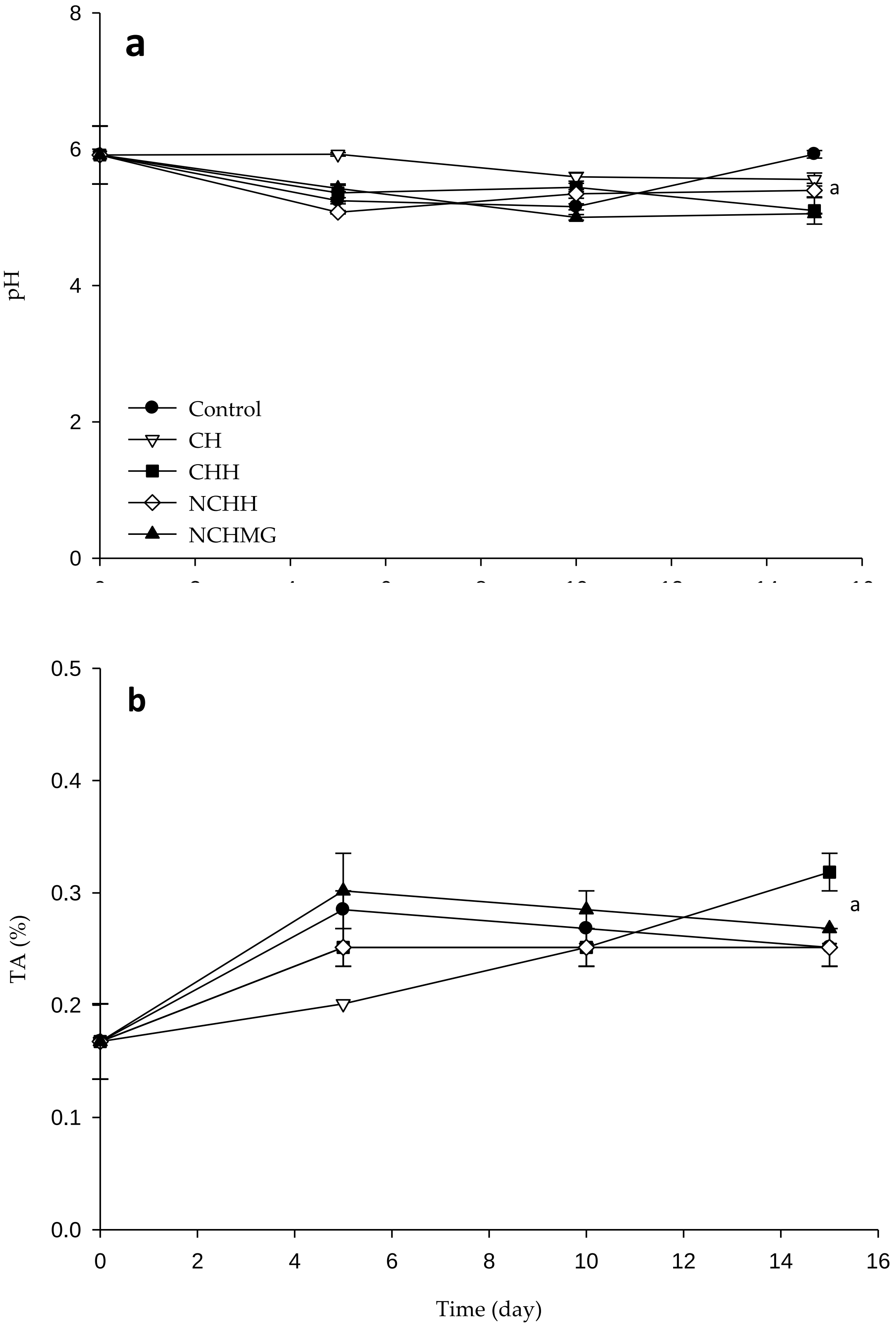

a
8
6
4
2
0
pH
a
Control
CH
CHH
NCHH
NCHMG
b
0.5
0.4
0.3
0.2
0.1
0.0
TA (%)
a
0
2
4
6
8
10
12
14
16
Time (day)

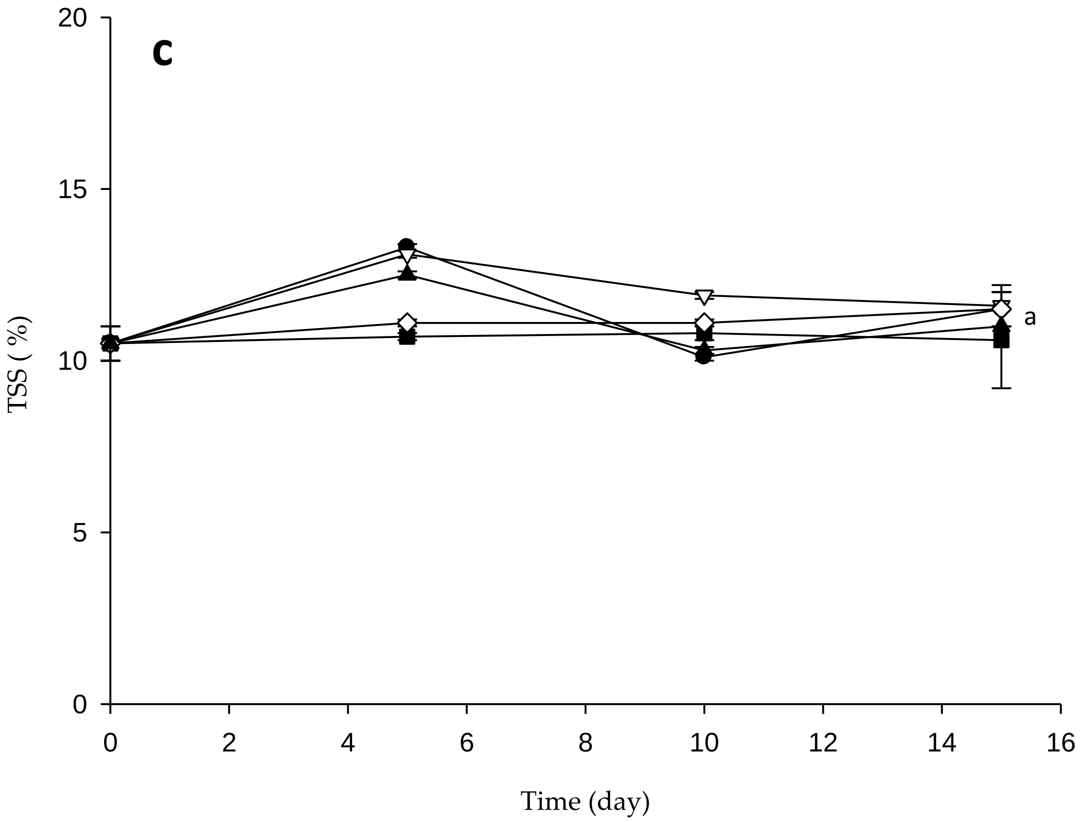

Control
Q
[illegible]

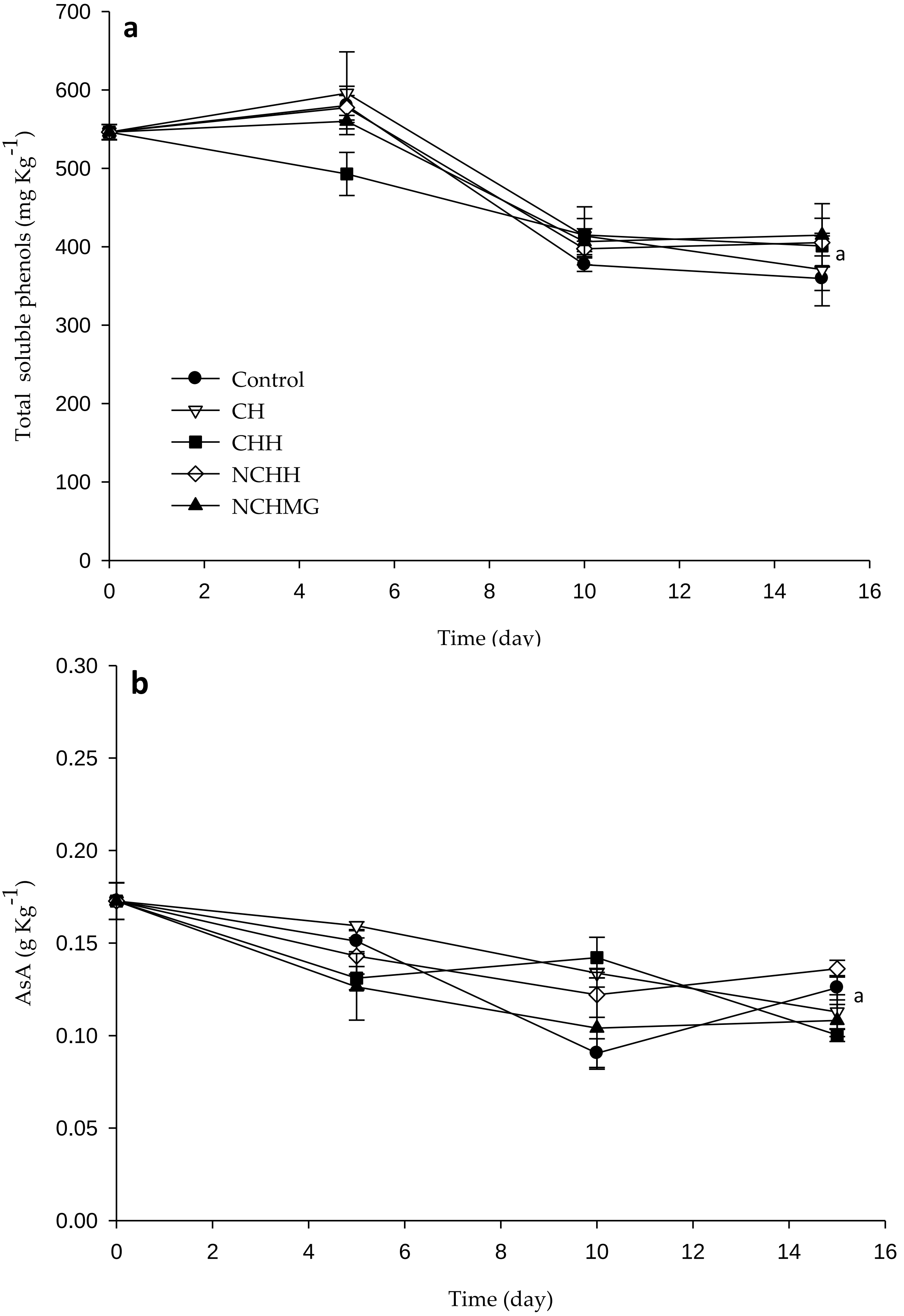

Figure 4. Phenolic compounds (a) and ascorbic acid (b) during storage at 10 °C and relative humidity (RH) of 80 % for fruit Control and coated samples with CH, CHH, NCHH, NCHMG. Data are the mean and their standard deviation (n=9). Different letters among traces mean significant differences ($p < 0.05$).

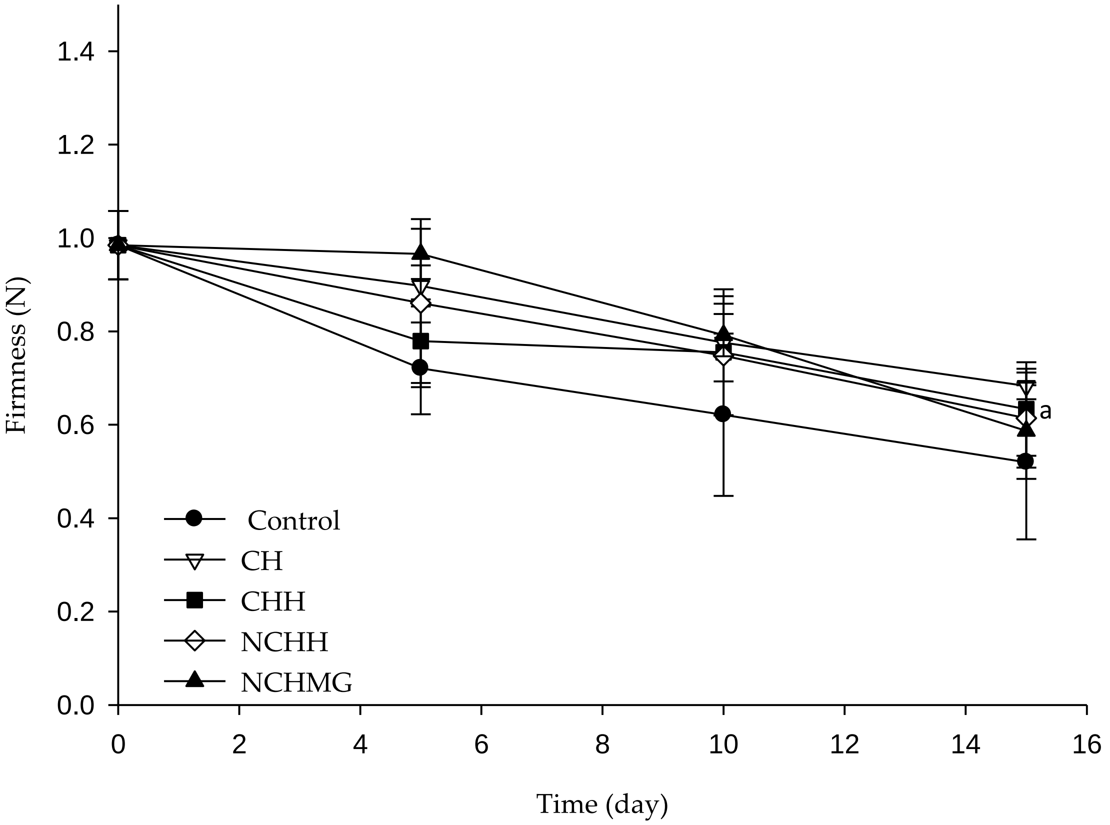

Figure 5. Firmness of pulp of pitayas determined during storage at 10 °C and relative humidity (RH) of 80% for fruit Control and coated samples with CH, CHH, NCHH, NCHMG. Data are the mean and their standard deviation (n=9). Different letters among traces mean significant differences ($p < 0.05$).

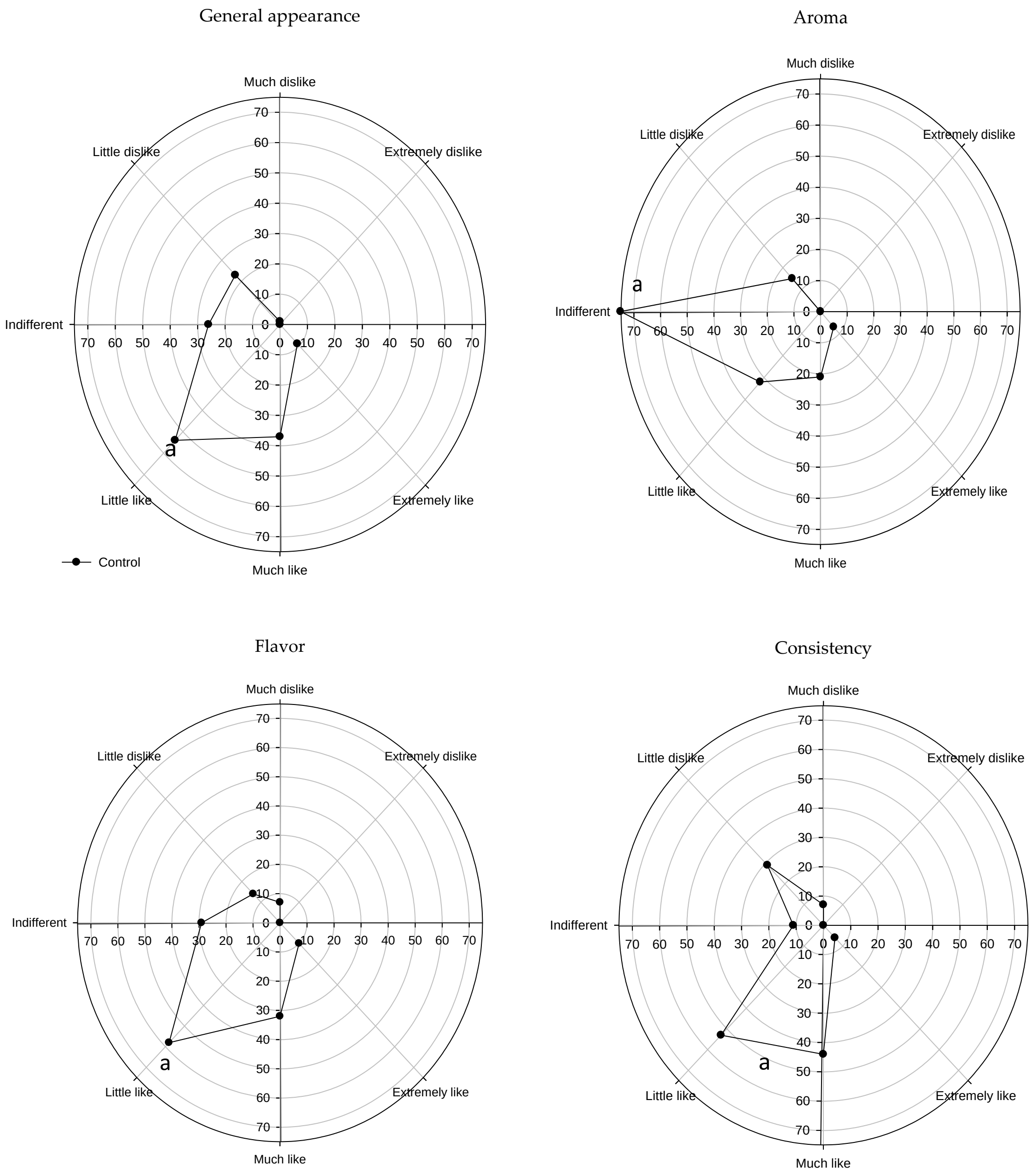

Figure 6. Sensory analysis of control fruit at the beginning of the bioassay (n=50).

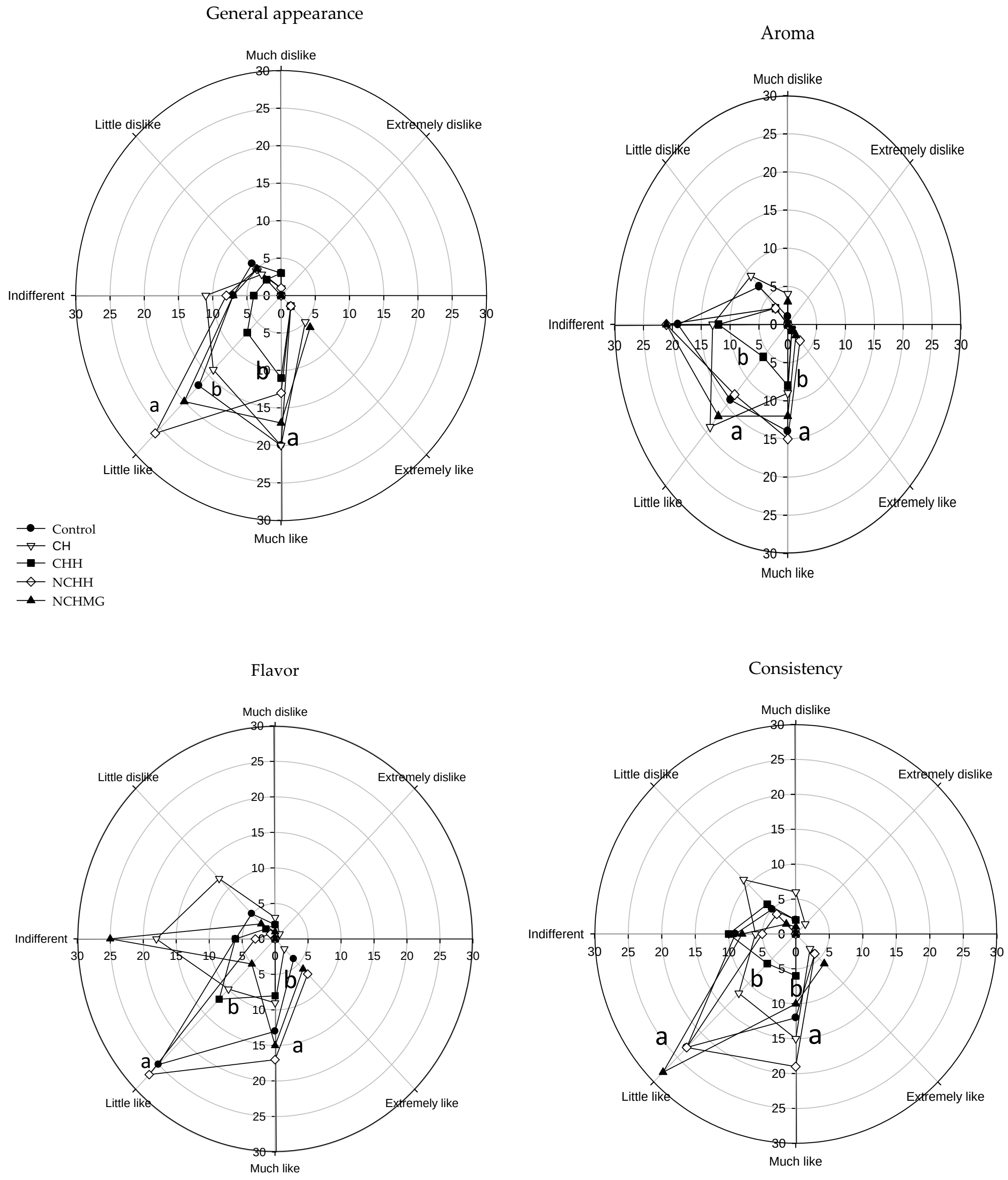

Figure 7. Sensory analysis of fruit: Control and coated samples with CH, CHH, NCHH, NCHMG stored at 10 °C and relative humidity (RH) of 80 % after 15 day (n=50).

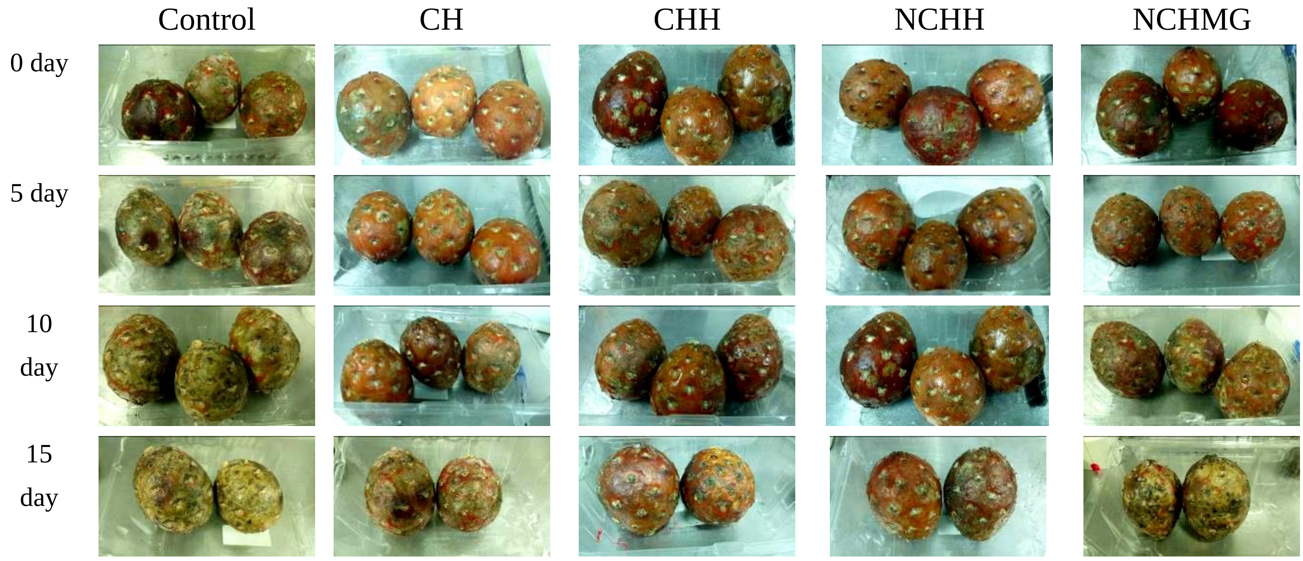

Figure S8: Photographs of pitayas control and coated with CH, CHH, NCHH, NCHMG after 0, 5, 10 and 15 day of storage at 10 °C and relative humidity (RH) of 80 %

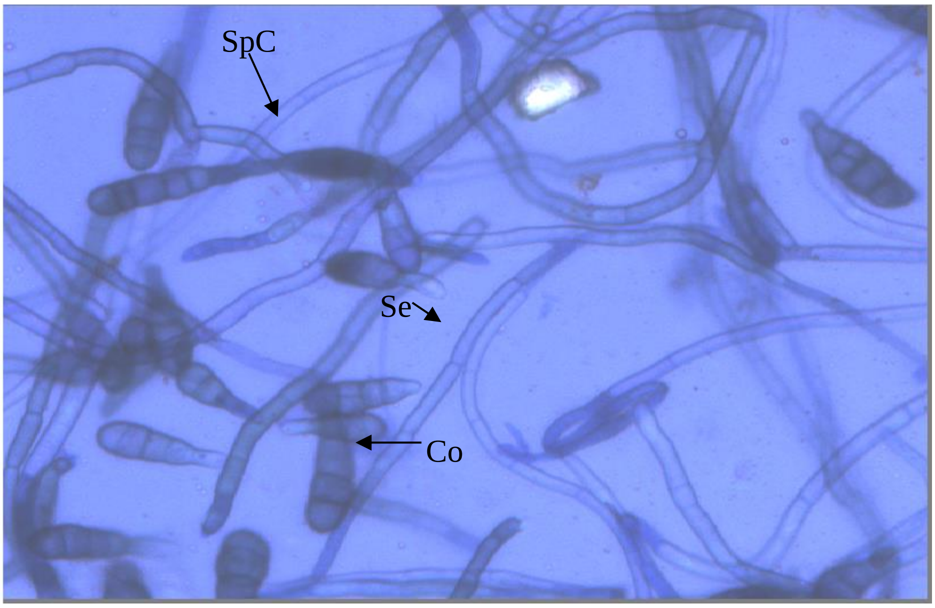

Figure 9. Photographs of light microscopy of fungal isolate stained with methylene blue at 100-magnification. Se is septate hyphae; Co is conidiophore; SpC is spore chain.

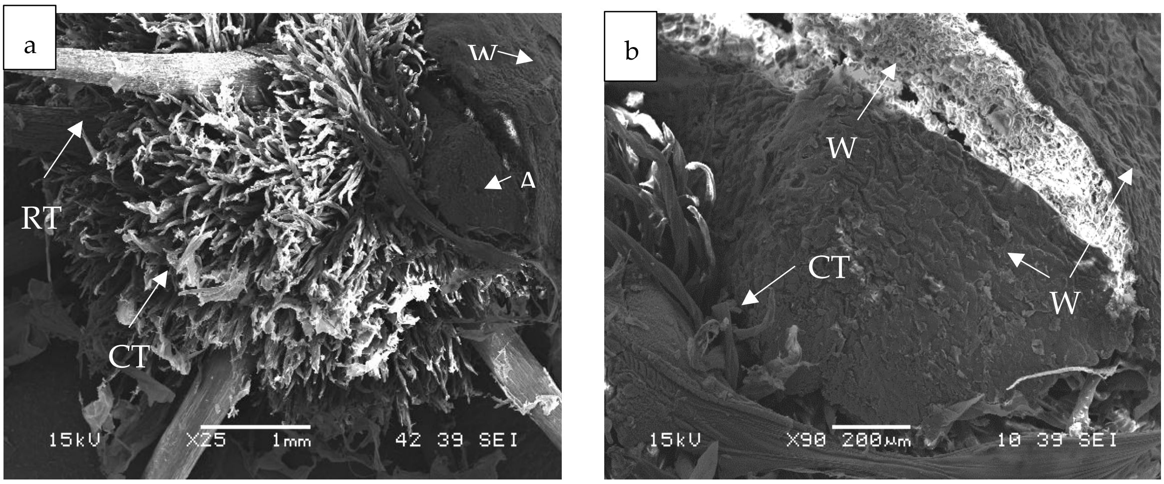

Figure 10. SE micrographs of the areola of pitaya (*S. pruinosus*). A is areola, RT is the radial spine, CT is the central spine, W is a natural wax, and WF is the wound after spine detachment (a, b).
